# Lymph node-targeted neoantigen nanovaccines potentiate anti-tumor immune responses of post-surgical melanoma

**DOI:** 10.1186/s12951-022-01397-7

**Published:** 2022-04-13

**Authors:** Yanhong Chu, Lingyu Qian, Yaohua Ke, Xiaoyu Feng, Xinjie Chen, Fangcen Liu, Lixia Yu, Lianru Zhang, Yaping Tao, Rui Xu, Jia Wei, Baorui Liu, Qin Liu

**Affiliations:** 1 The Comprehensive Cancer Centre of Nanjing Drum Tower Hospital, The Affiliated Hospital of Nanjing University Medical School, 321 Zhongshan Road, Nanjing, 210008 China; 2 Department of Oncology, Rudong Peoples’ Hospital of Jiangsu Province, Nantong, China; 3 Department of Pathology, Affiliated Nanjing Drum Tower Hospital of Nanjing University Medical School, Nanjing, China

**Keywords:** Neoantigen, Cancer vaccine, Nanoparticle, Immunotherapy, Anti-PD1 antibody

## Abstract

**Background:**

Neoantigens are considered ideal targets for immunotherapy, especially tumor vaccine, because of their strong specificity and immunogenicity. Here, we developed a neoantigen nanovaccine used liposomes with lymph-node targeting characteristic.

**Methods:**

Our nanovaccine was composed of neoantigens, an amphiphilic liposome and an adjuvant Montanide™ ISA 51. Small animal imaging system and immunofluorescence staining were used to identify the distribution of nanovaccines. A subcutaneous-tumor-resection mouse model of melanoma was established to evaluate the anti-tumor efficacy. Flow cytometry was performed to assay the immune responses initiated by nanovaccines.

**Results:**

Nanovaccines could traffic to lymph nodes, be uptaken by CD11c^+^ DCs and promote DCs maturity. After the treatment of our neoantigen nanovaccines, the average recurrence time was extended from 11 to 16 days and the median survival time was even prolonged 7.5 days relative to the control group (NS group). Nanovaccines increased neoantigen-specific T cells to 10-fold of free vaccines, and upregulated Th1 cytokines, such as IFN-γ and TNF-α. The anti-tumor activity of spleen lymphocytes in the nanovaccine group was significantly stronger than that of other groups. However, some immune-inhibitory cells or molecules in tumor microenvironment have been detected upregulated under the immune pressure of neoantigen nanovaccines, such as Tregs and PD-L1. The efficacy of the neoantigen nanovaccine combined with anti-PD1 antibody or Treg inhibiting peptide P60 was better than that of the single treatment.

**Conclusions:**

We developed a general vaccine strategy, triggering specific T cell responses, and provided feasible combination strategies for better anti-tumor efficacy.

**Graphical abstract:**

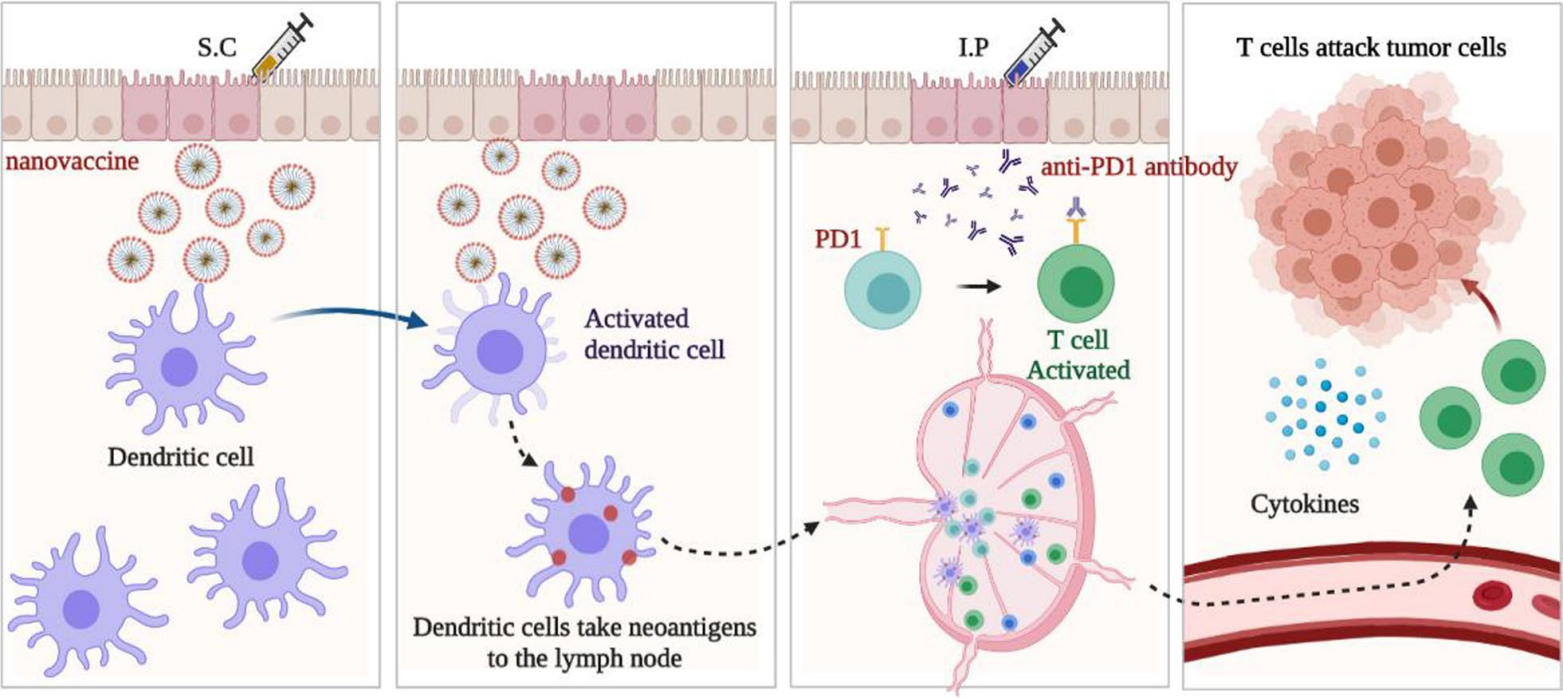

**Supplementary Information:**

The online version contains supplementary material available at 10.1186/s12951-022-01397-7.

## Introduction

Melanoma is the most lethal skin cancer and the efficacy of immune checkpoint inhibitors (ICIs) on it is not satisfactory. Patients with metastatic melanoma had an objective response rate (ORR) of only 10.9% with ipilimumab [1] and a 5-year survival rate of 41% with pembrolizumab [2]. To improve the efficacy of immunotherapy or overcoming drug resistance, the strategies include changing ‘cold’ tumors (low mutation load and less tumor infiltrating lymphocytes) into ‘hot’ tumors, eliminating immunosuppressive factors and so on. At present, the cancer vaccine is an active area, can induce and amplify tumor-specific T cell responses and form long-term immune memory, providing bright prospect of clinical application [3]. However, only one therapeutic vaccine ‘Provenge’ has been approved by USA food and drug administration (FDA) for the treatment of prostate cancer, and many cancer vaccine clinical trials have modest efficacy [4].

As we previously reviewed, selection of antigens is the key factor of the clinical efficacy of cancer vaccines [5]. Neoantigens, produced by mutant proteins or oncogenic viruses integrated into the genome, without thymus negative screening and only expressed in tumor tissues, are currently recognized as the ideal targets for cancer vaccines [6]. With the development of next-generation sequencing and bioinformatics, it is possible to identify neoantigens for individuals. Personalized neoantigen vaccines have begun to achieve good efficacy in small-scale early clinical trials [7–9].

Most antigens utilized by vaccines are proteins, peptides or nucleic acids, which have poor stability in vivo. In order to play an anti-tumor role, vaccines need to be effectively delivered to secondary lymphoid organs, in which immune responses mainly occur. The nanomaterial carrying both tumor antigens and adjuvants, is one of the most common and successful methods to activate antitumor immune responses, due to the ability to target tumors, lymph nodes, or antigen presenting cells (APCs). In addition, the protective effect of nanomaterials on loaded drugs makes the types of antigens available for tumor vaccines more abundant [10]. In general, the immune system uses major histocompatibility complex (MHC) class II pathway to eliminate extracellular soluble non-selfantigens [11]. Different from extracellular soluble antigens, the antigens on extracellular nanoparticles often undergo a cross-presentation process after being ingested by APCs, and are loaded on MHC I and presented to CD8^+^ T cells, the main force to kill tumor cells [12, 13].

Therapeutic cancer vaccines still face unique tumor microenvironment challenges. Solid tumors often obtain immune escape, grow and metastasize rapidly by inducing hypoxia and low pH in the microenvironment, expressing immunosuppressive molecules, such as programmed cell death protein 1 ligand 1 (PD-L1) and cytotoxic T-lymphocyte-associated protein 4 (CTLA-4), and recruiting immunosuppressive cells, such as regulatory T cells (Tregs) and myeloid-derived suppressor cells (MDSCs). ICIs are monoclonal antibodies against checkpoint proteins expressed by immune cells or tumor cells, which can reactivate T cell responses by blocking immunosuppressive signal pathways [14–16]. More and more evidence supports the view that if tumor patients lack pre-existing tumor infiltrating lymphocytes (TILs), they are unlikely to benefit from ICIs treatment [17, 18]. Based on the abilities of cancer vaccines to induce and amplify TILs, therapeutic vaccines and ICIs may have synergistic antitumor effect. Treatments against immunosuppressive cells can also be considered as potential targets for vaccination. For example, P60, a short peptide, can inhibit Tregs function by binding to Forkhead box protein P3 (Foxp3) [19].

In the absence of inflammation and/ or microbial stimulation, the antigen presented by dendritic cells (DCs) in a stable state will induce immune tolerance rather than activate immunity, indicating that the antigen administered alone is a weak inducer of adaptive immunity. So effective vaccination requires additional immune adjuvants [11]. Montanide™ ISA 51 is an oil-in-water emulsion immune adjuvant. It can keep in the injection site and attract APCs to capture and process vaccine antigens [20–22]. In a clinical trial, patients with high-risk vulvar intraepithelial neoplasia (precancerous lesions) were inoculated with the long peptides of HPV E6 and E7 mixed with Montanide™ ISA 51. This vaccine induced CD4^+^ and CD8^+^ T cell responses targeting these two antigens, and 47% of patients had complete clinical responses [20].

In this study, we developed a novel amphiphilic neoantigen nanovaccine, composed of neoantigens and a polyethylene glycol phospholipid derivative approved by USA FDA, mixed with an adjuvant Montanide™ ISA 51. Here, we verified its lymph node-targeted characteristic, evaluated anti-tumor effect and safety of the neoantigen nanovaccine in the mouse model of preventing recurrence and metastasis after subcutaneous tumor resection of melanoma and examined neoantigen-specific T cell immune responses elicited by the nanovaccine. Furthermore, we explored some strategies to improve the efficacy of our neoantigen nanovaccine, including immune checkpoint blockade as well as Treg inhibiting peptide P60.

## Materials and methods

### Mice and cell lines

C57BL/6 mice aged 6–8 weeks were purchased from Changzhou Cavens laboratory animal Co. Ltd. (Changzhou, China). All mice were kept in the specific pathogen-free (SPF) Laboratory Animal Center of Affiliated Nanjing Drum Tower Hospital of Nanjing University Medical School (Nanjing, China). All animal experimental protocols were approved by the Laboratory Animal Care and Use Committee of the Affiliated Nanjing Drum Tower Hospital of Nanjing University Medical School.

B16F10 melanoma cells were purchased from the Cell Bank of the Chinese Academy of Sciences (Shanghai, China) and cultured in RPMI 1640 medium containing 10% fetal bovine serum (FBS) at 37 °C under an atmosphere of 5% CO_2_.

### Preparation and characterization of nanovaccines

DSPE-PEG_2000_-NHS (Xi’an ruixi Biological Technology, China) and antigen peptide (Tyrp1: TAPDNLGYM, M20: FLHWYTGEAMDEMEFTEAE, M27: LCPGNKYEM, synthesized by ChinaPeptides, China) were mixed in phosphate buffer saline (PBS) at pH 7.4 in a molar ratio of 1:1 and vibrated at room temperature for 24 h. The product DSPE-PEG_2000_-peptide was dialyzed overnight and the dialysate was changed every 2 h for at least 3 times to remove unreacted DSPE-PEG_2000_-NHS and peptide. The obtained product was freeze-dried and then verified by ^1^H NMR (Bruker, German) and MALDI-TOF-MS (Bruker, German).

DSPE-PEG_2000_-peptide freeze-dried powder was dissolved in PBS or ddH_2_O to form nanoparticles. The particle size and zeta-potential of nanoparticles were detected by a particle-size potentiometer (Malvern, UK). The size and morphology of nanoparticles were observed by transmission electron microscope (TEM, JEOL, Japan) without negative staining.

The encapsulation efficiency and drug loading content of DSPE-PEG_2000_-peptide nanoparticles were determined by high performance liquid chromatography (HPLC). For each peptide, the detection template was set according to the HPLC report provided by the production company. (column temperature: 25 ℃, injection quantity: 10 μl, velocity of flow: 1 ml/min, mobile phase ratio: 1/1000 TFA Acetonitrile: 1/1000 TFA water = 20:80–61:39 12 min Tyrp1, 25:75–60:40 20 min M20; 15:85–43:57 17 min M27) The peak areas of free peptide at different concentrations (500 μg/ml, 250 μg/ml, 125 μg/ml, 62.5 μg/ml, 31.25 μg/ml) were tested to draw the standard curve. The drug loading content and encapsulation efficiency can be obtained according to the following calculation formula.

$${\text{Drug loading content}}\% =\frac{\text{Weight of the drug in nanoparticles}}{\text{Weight of the nanoparticles}} \times 100\%$$

$${\text{Encapsulation efficiency\% = }}\frac{{\text{Weight of the drug in nanoparticles}}}{{\text{Weight of the feeding drug}}} \times 100\%$$

### In vitro release of peptide from nanovaccines

Equivalent DSPE-PEG_2000_-peptide was dissolved respectively in 1 ml PBS and 0.5 ml PBS + 0.5 ml adjuvant Montanide™ ISA 51 (Seppic, France). The two samples were placed in a 2000 Da dialysis bag and dialyzed with double distilled water (dialysate). Detect the peptide concentration in dialysate by HPLC at 6 h, 24 h, 48 h, 72 h, 96 h, 120 h and 144 h.

### In vivo distribution of nanovaccines

Peptides were labeled with dye NIR797 (Xi’an ruixi, China), and then DSPE-PEG_2000_-peptide-NIR797 nanoparticles were prepared according to the above method. Equivalent peptide-NIR797 and DSPE-PEG_2000_-peptide-NIR797 were separately injected subcutaneously at the tail base of mice. Mice were anesthetized with isoflurane and imaged by an optical and X-ray small animal imaging system (IVIS Lumina, Perkin Elmer, German) at 2 h, 24 h and 48 h respectively. Then, mice were sacrificed and inguinal Lymph nodes, spleens and livers were excised and imaged. Image analysis: ROI was used to delineate specific areas to obtain the average fluorescence intensity.

### Immunofluorescence staining

A model antigen ovalbumin (OVA) was used to evaluate the distribution of nanovaccines. DSPE-PEG_2000_-OVA-FITC nanoparticles (OVA-FITC: CSSIINFEK- FITC) were prepared according to the above method. Inguinal lymph nodes were obtained 48 h after immunization, made into frozen sections, and incubated with anti-CD3 rat monoclonal antibody (1:200) (Abcam, UK), anti-CD11c rabbit monoclonal antibody (1:200) (Cell Signaling Technology, USA) overnight at 4 °C. After washing with PBS 3 times, the sections were stained with goat anti-rabbit IgG H&L (Cy3, 1:200) (Abcam, UK), goat anti-rat IgG H&L (Cy5, 1:200) (Abcam, UK) and DAPI (Sangon Biotech, China). After sealed with 50% glycerol, fluorescence images were taken by confocal microscope (Leica, German).

### Nanovaccines activate BMDCs in vitro

Bone marrow derived dendritic cells (BMDCs) of C57BL/6 mice were cultured with 20 ng/mL GM-CSF (Peprotech, USA) and 10 ng/mL IL-4 (Peprotech, USA). Media was replaced on day 3; non-adherent and loosely adherent immature dendritic cells (iDCs, routinely 60–80% CD11c^+^) were collected on day 6. Then, iDCs were incubated with free peptide (Tyrp1, M20 or M27, 10 μg/ml) and equivalent nanoparticles (Tyrp1-NP, M20-NP or M27-NP). Cells were collected 48 h later and stained with CD11c-FITC, CD80-APC and CD86-PE antibodies (Biolegend, USA). BD Accuri C6 (BD Bioscience, USA) were used to detect activated DCs.

### Animal experiments

B16F10 melanoma cells were subcutaneously injected to the left lower abdomen of C57BL/6 mice (2 × 10^5^ cells per mice, D0). About 3 days later, when the tumor size was almost 50 mm^3^, the tumor was surgically removed (surgical margin: tumor-cell positive). Penicillin was injected intramuscularly for 3 days after surgery (2 × 10^4^ UI per mouse). After that, mice were randomly divided into four groups: normal saline (NS, 100 μl per mouse), vehicle (DSPE-PEG_2000_-NHS), free vaccine (Tyrp1 + M20 + M27, each peptide 20 μg dissolved in 50 μl NS and mixed with 50 μl adjuvant Montanide™ ISA 51), nanovaccine (equivalent nanoparticles prepared from the above three antigens, mixed with Montanide™ ISA 51). Vaccine treatment was started on D6, once every other day, five times in total. The tumor sizes and body weights were measured once every two or three days. Mice were euthanized when the length diameters of tumors reached 1.5 cm. On D20, mice were taken MRI pictures. On D21, hearts, livers, lungs and kidneys were excised for hematoxylin–eosin staining (HE staining), and spleens, lymph nodes and tumors were collected for flow cytometry.

Combination therapy of vaccine and systemic anti-programmed cell death protein 1(PD1) antibody: anti-PD1 antibody (100 μg per mouse) was intraperitoneally injected on D8, D10 and D12. Murine anti-PD1 antibody (G4C2) was provided by Shanghai Junshi Biosciences Co., Ltd (Suzhou, China).

Combination therapy of nanovaccine and P60 (RDFQSFRKMWPFFAM): DSPE-PEG_2000_-P60 nanoparticles (P60-NP) were prepared according to the above method. P60-NP treatment (P60: 20 μg per mouse) procedure was the same as neoantigen nanovaccinestreatment, started on D6, once every other day, five times in total.

### Flow cytometry

Single cell suspension preparation: lymph nodes and spleens were ground, filtered and suspended in NS (0.5–1 × 10^6^ cells/ml); tumors were cut into small pieces, incubated with collagenase type IV (1 mg/ml, Sigma, USA) at 37 ℃ for 3–4 h, filtered and suspended in NS (0.5–1 × 10^6^ cells/ml). Most tested antigens are expressed on cell membranes. In this situation, samples were stained with specific antibodies for 20 min in 4 °C in dark, and then washed before analysis. Specially, the True-Nuclear Transcription Factor Buffer Set (Biolegend, USA) was used to test Foxp3, which is expressed in nucleus. The following monoclonal antibodies (mAbs) were used for flow cytometry and purchased from Biolegend: CD11c-FITC (5 μg/ml), CD80-APC (2 μg/ml), CD86-PE (2 μg/ml), CD3-FITC (5 μg/ml), CD4-PE-Cy7 (2 μg/ml), CD8-PE-Cy5 (2 μg/ml), CD44-PE (2 μg/ml), CD62L-APC (2 μg/ml), PD-L1-PE (2 μg/ml), Foxp3-PE (2 μg/ml).

To detect the frequency of M27-specific CD8^+^ T cells by pMHC multimer staining, cells were incubated with anti-mouse CD16/CD32 (BD Biosciences, 10 μg/ml) for 30 min at 4 °C to block Fc receptors, then incubated with a mixture of H-2 Kb/M27-Ig dimers (BD Biosciences, 5 μg/ml) and APC-anti-mouse IgG1 (BD Biosciences, 5 μg/ml) for 1 h at 4 °C in the dark. After washing, CD3-FITC and CD8-PE-Cy5 were added for an additional 30 min at 4 °C and followed by flow cytometry.

BD™ Cytometric Bead Array (CBA) Mouse Interleukin (IL)-6 Flex Set, Mouse Interleukin (IL)-6 Flex Set and Mouse Th1/ Th2 Cytokine Kit were used to detect and analyze the level of IL-6, IL-10, IL-2, IL-4, IL-5, IFN-γ, TNF-α.

### Statistical analysis

For all experiments, biological replicates were performed unless otherwise stated. One-way ANOVA with Tukey’s multiple comparisons test or two-way ANOVA with Tukey’s HSD multiple comparison post hoc test was performed to compare several groups. Survival benefit was determined with the Log-rank (Mantel-Cox) test. All statistical analysis and statistical charts are completed by Graphpad Prism 8.0 software. The data were expressed as mean ± standard deviation (mean ± SD) or mean ± standard error (mean ± SEM). *P* < 0.05 showed statistical significance.

## Results

### Synthesis and characterization of neoantigen nanovaccines

**Fig. 1:**
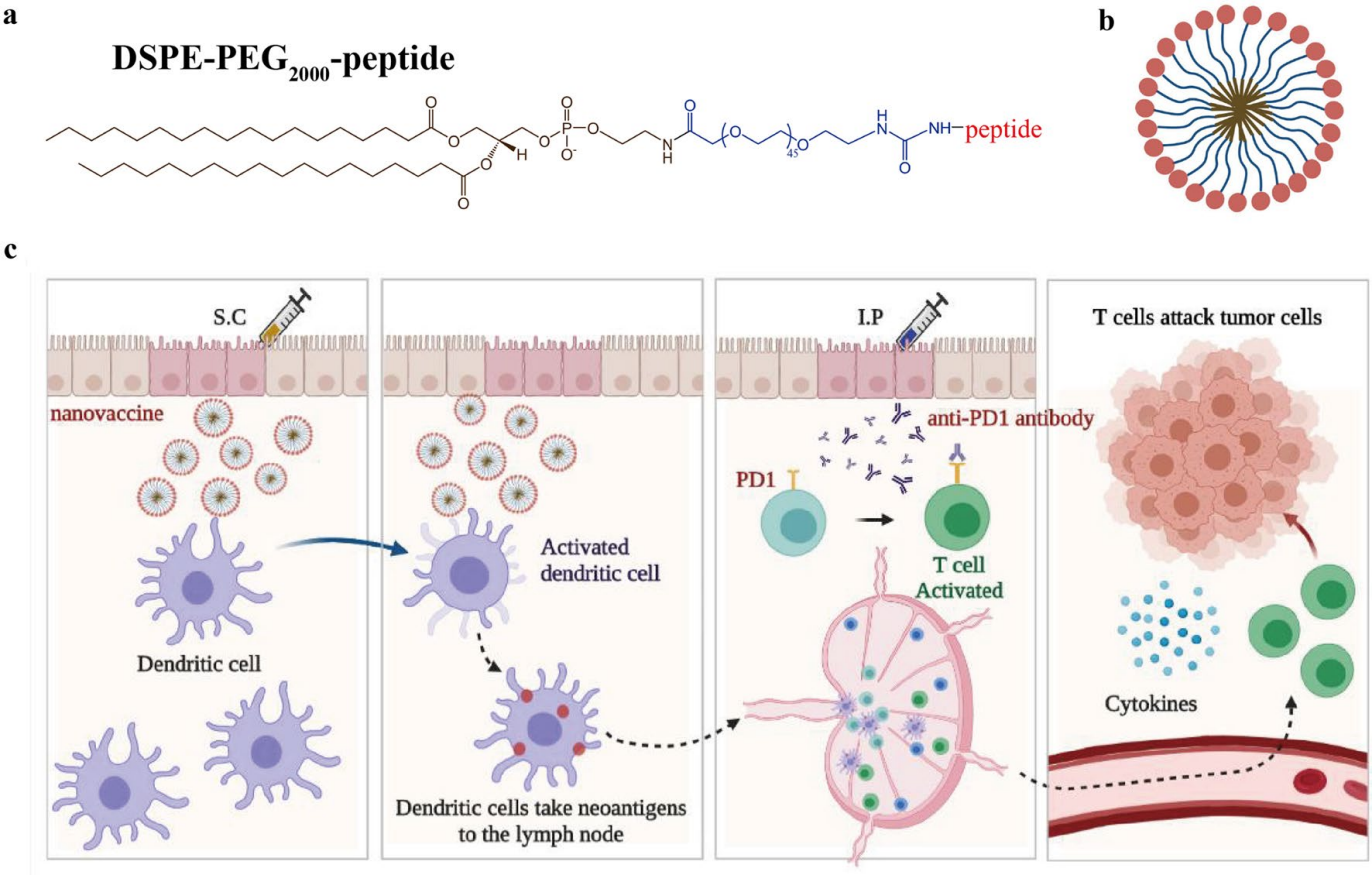
Design of the neoantigen nanovaccine. **a** Chemical structural formula of DSPE-PEG_2000_-peptide. **b** Pattern diagram of a nanoparticle.Brown lines gathered together in the center represent DSPE, blue lines represent PEG_2000_, and red circles represent various peptides. **c** Pattern diagram of the neoantigen nanovaccine treatment combined with anti-PD1 antibody

We synthesized an amphiphilic nanoparticle (DSPE-PEG_2000_-peptide) by additive reaction of DSPE-PEG_2000_-NHS and peptide (two neoantigens of B16F10 melanoma cells: M20 and M27, with a highly-expressed peptide Tyrp1). Figure 1a shows its chemical structural formula. When DSPE-PEG_2000_-peptide is dissolved in PBS, hydrophobic DSPE ends will gather together to form nanoparticles (Fig. 1b). We developed a neoantigen nanovaccine based on this nanoparticle and adjuvant Montanide™ ISA 51, combined with anti-PD1 antibody, to trigger and amplify tumor specific T cell responses (Fig. 1c).

**Fig. 2:**
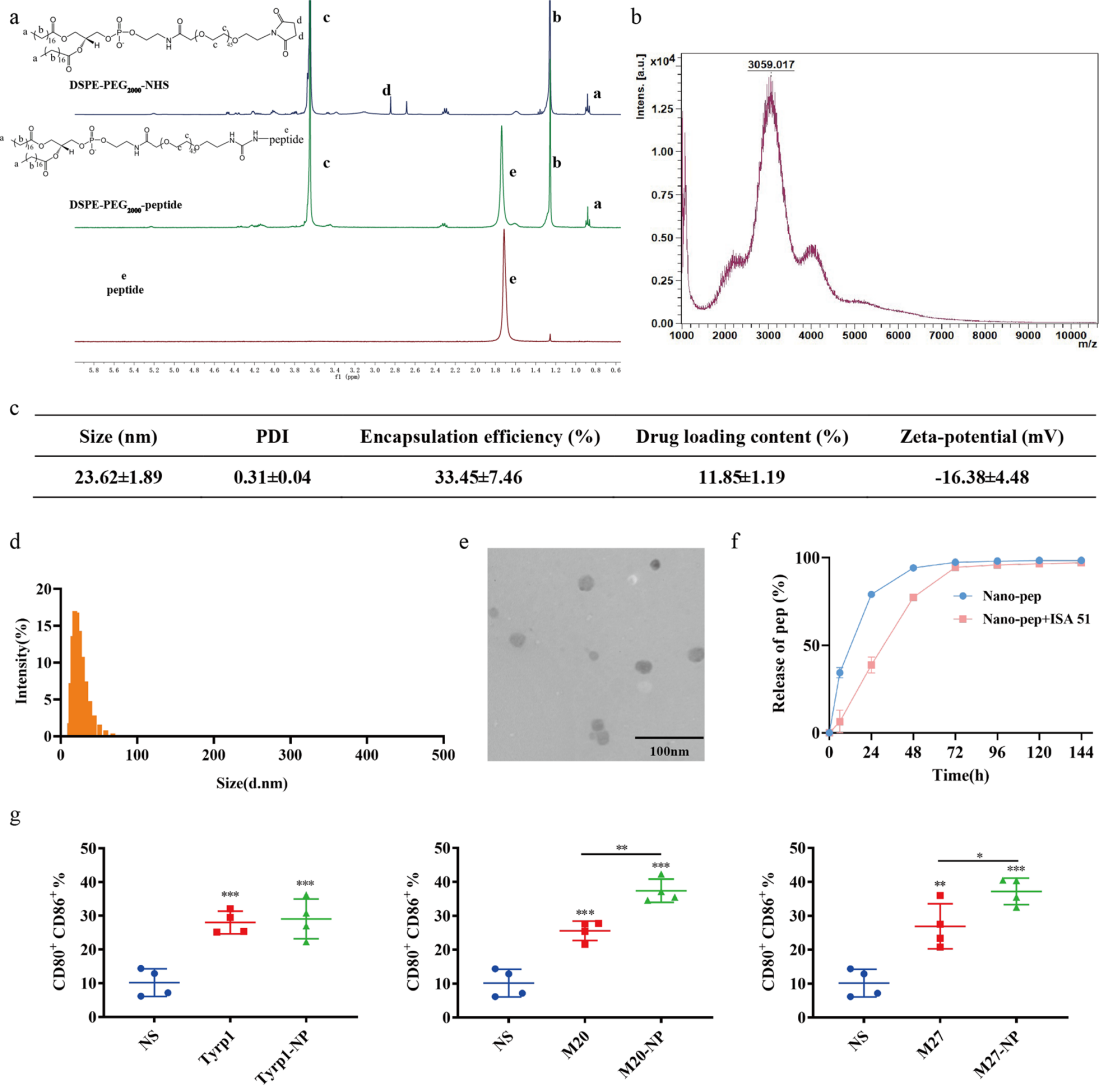
Characterization and in vitro DC-activation of neoantigen nanovaccines. **a** ^1^H NMR of DSPE-PEG_2000_-NHS (vehicle), DSPE-PEG_2000_-peptide and peptide (peptide: M27). **b** MALDI-TOF–MS of DSPE-PEG_2000_-peptide (peptide: M27). **c** Size, PDI, encapsulation efficiency, drug loading content and zeta-potential of nanovaccines. **d** Size of nanovaccines. **e** The transmission electron microscopy (TEM) image of nanovaccines. **f** Curves of peptide (M27) release from nanovaccines in different solutions. **g** Proportion of mature DC (CD11c^+^CD80^+^CD86^+^) after incubation with normal saline (NS), peptide (Tyrp1, M20 or M27) or nanovaccines (Tyrp1-NP, M20-NP or M27-NP) for 48 h. *P*-values were determined by one-way ANOVA with Tukey’s multiple comparisons test. **P = 0.0026 (M20 vs M20-NP), **P = 0.0029 (NS vs M27), *P = 0.0429 (M27 vs M27-NP), ***P < 0.001

The synthetic DSPE-PEG_2000_-peptide was verified by ^1^H NMR and MALDI-TOF–MS (Fig. 2a, b, Additional file 1: Fig. S1a). It is known that the molecular weight of DSPE-PEG_2000_-NHS is about 2000 Da and that of peptide (M27, 9-mer) is about 1000 Da. The molecular weight of DSPE-PEG_2000_-peptide shown in Fig. 2b is about 3000 Da, which is consistent with the expected value. The encapsulation efficiency and drug loading of nanovaccines were determined by HPLC, and the average values were 33.45 ± 7.46% and 11.85 ± 1.19% respectively (Fig. 2c, Additional file 1: Fig. S1b). The size of nanovaccines was about 24 nm (Fig. 2c–d, Additional file 1: Fig. S1b). The TEM image also shows that the morphology of nanovaccines was spherical with a diameter of 20–30 nm (Fig. 2e). Nanovaccines completely released the peptide (M27) in PBS for 48 h, while it took 72 h to fully release the peptide in PBS mixed with adjuvant Montanide™ ISA 51 (Fig. 2f).

### In vitro DCs-activation of neoantigen nanovaccines

BMDCs of C57BL/6 mice were cultured and incubated with these three peptides or equivalent nanovaccines, after stimulation with GM-CSF and IL-4. The proportion of mature DCs (CD11c^+^CD80^+^CD86^+^) was significantly upregulated compared with NS group (Fig. 2g). Moreover, for the two neoantigens, M20 and M27, the proportion of mature DCs in the nanovaccine group was 1.5-fold higher than that in the free peptide group.

### Lymph node-targeting characteristic of neoantigen nanovaccines

**Fig. 3:**
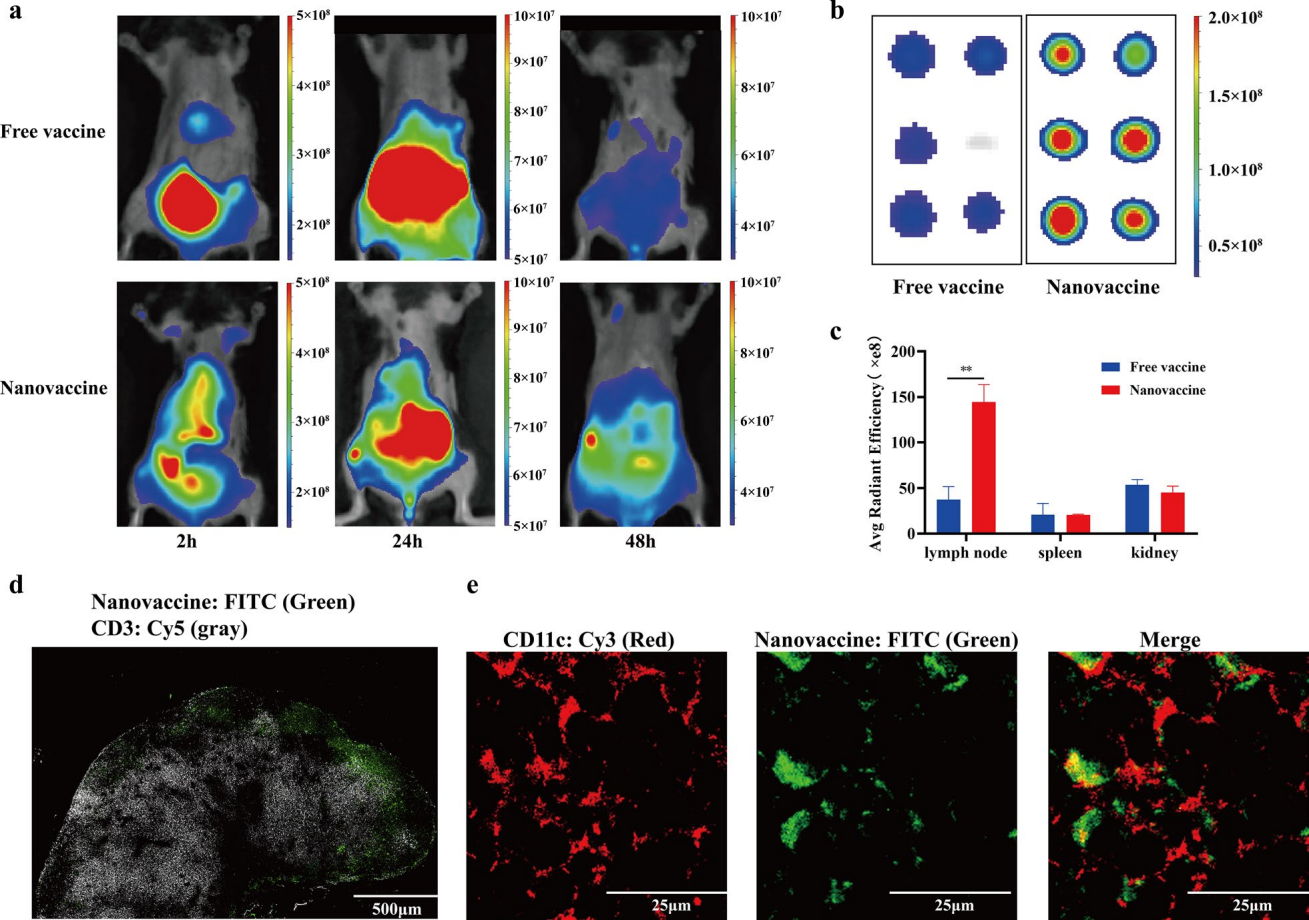
Lymph node-targeting characteristic of neoantigen nanovaccines. **a** Equivalent peptide-NIR797 (Free vaccine group) and DSPE-PEG_2000_-peptide-NIR797 (Nanovaccine group) were separately mixed with Montanide™ ISA 51 and injected subcutaneously at the tail base of mice. The fluorescence distribution in mice at different time points was photographed by small animal in vivo imaging (n = 3). **b** Fluorescence image of inguinal lymph nodes 48 h after injection. **c** The average radiant efficiency of inguinal lymph nodes, spleens and kidneys 48 h after injection. *P*-values were determined by one-way ANOVA with Tukey’s multiple comparisons test. **P = 0.0062. **d**–**e** A FITC labeled model antigen ovalbumin (OVA) was used to evaluate the distribution of nanovaccines. **d** Localization of nanovaccines and CD3^+^ T cells in inguinal lymph nodes 48 h after subcutaneous injection of DSPE-PEG_2000_-OVA-FITC, was shown by immunofluorescence staining. Nanovaccine: green (FITC); T cells (CD3): gray (Cy5); Scale: 500 μm. **e** Localization of nanovaccines and DCs in lymph nodes 48 h after subcutaneous injection of DSPE-PEG_2000_-OVA-FITC. Nanovaccine: green (FITC); DCs (CD11c): red (Cy3); Scale: 25 μm

Free vaccines (peptide-NIR797) and nanovaccines (DSPE-PEG_2000_-peptide- NIR797) were separately mixed with Montanide™ ISA 51 and injected subcutaneously at the tail base of mice. Fluorescence photos taken at 2 h, 24 h and 48 h showed that although the fluorescence signal of the lymph node area was declining with the passage of time, the signal in nanovaccine group was stronger than that in Free vaccine group at each time point (Fig. 3a). After 48 h, mice were sacrificed and inguinal lymph nodes, spleens and livers were excised and imaged. Nanovaccines showed a 2.5-fold greatly enhanced lymph-node accumulation compared with that of free vaccines (Fig. 3b). In addition, no significant difference of vaccine accumulation was found in spleens and livers (Fig. 3c).

Inguinal lymph nodes were excised and made into frozen sections 48 h after subcutaneous injection of DSPE-PEG_2000_-OVA-FITC nanovaccines. Figure 3d shows the distribution of T cells (CD3-gray) and nanovaccines (FITC-green) in lymph nodes. Nanovaccines can be seen in the T-cell gathering area. There is co-localization between DCs (CD11c-red) and nanovaccines (FITC-green), suggesting that nanovaccines can be uptaken by DCs in lymph nodes (Fig. 3e).

### Anti-tumor efficacy and safety of neoantigen nanovaccines

**Fig. 4:**
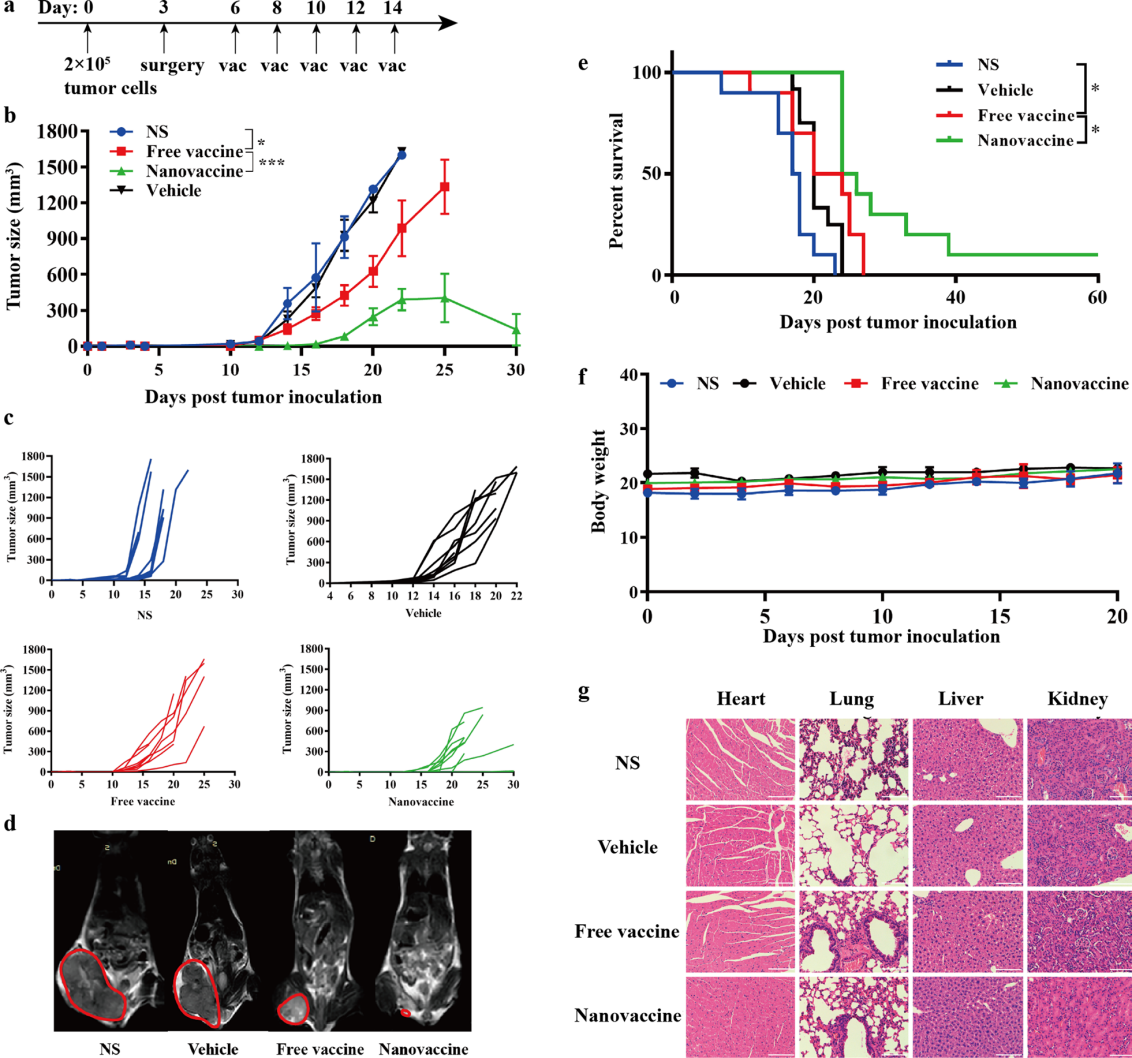
Anti-tumor efficacy and safety of neoantigen nanovaccines. **a** Treatment schema of neoantigen nanocaccines. C57BL/6 mice were implanted with B16F10 melanoma cells (2 × 10^5^) on the left lower skin of abdomen on Day 0. About 3 days later, when the tumor size was almost 50 mm^3^, the tumor was surgically removed (surgical margin: tumor-cell positive). Mice received treatments on Day 6, 8, 10, 12 and 14. **b** Growth curves represent the average tumor volumes of each group. (n = 10). **c** Tumor growth curves of each mouse in different groups. (n = 10). *P*-values were determined by two-way ANOVA with Tukey’s HSD multiple comparison post hoc test. *P = 0.0115 (NS vs Free vaccine), ***P < 0.001. **d** MRI images of representative mice were taken on Day 20. The tumor of each mouse was circled. **e** Survival curves. (n = 10). *P*-values were determined by Log-rank (Mantel-Cox) test. *P = 0.0135 (NS vs Free vaccine), *P = 0.0254 (NS vs Free vaccine). **f** Body weights of mice in each group. (n = 10). **g** Hematoxylin–eosin staining of main organs, including heart, lung, liver and kidney. Scale: 100 μm. *P*-value: *, *P* < 0.05; ***, *P* < 0.001

In order to verify the efficacy of this melanoma neoantigen nanovaccine, we established a recurrence-prevention mouse model. After tumor resection, mice were randomly divided into four groups: NS group, vehicle group, free vaccine group and nanovaccine group. The treatment scheme is shown in Fig. 4a. Figure 4b and c show that the tumor recurrence and growth of mice in NS group and vehicle group are quite rapid. The tumor growth of mice in Free vaccine group is slightly slower than that of the first two groups. The average recurrence time in nanovaccine group is about 4 days longer than that of free vaccine group (16 vs 12, *p* = 0.0014), and the tumor growth is significantly inhibited. MRI images of mice in each group on the 20th day post tumor inoculation, also shows that tumors in the nanovaccine group are the smallest at this time point (Fig. 4d). Accordingly, the median survival time of mice in the nanovaccine group was also prolonged 7.5 days than that of NS group (*p* < 0.001), and 3 days that of free vaccine group (*p* = 0.0254, Fig. 4e).

The trend of body weights in each group kept stable during treatment (Fig. 4f). Hearts, lungs, livers and kidneys were excised and made into paraffin sections, one week after the last treatment. After HE staining, no obvious abnormality was found in all sections (Fig. 4g).

### T cell responses activated by neoantigen nanovaccines

**Fig. 5:**
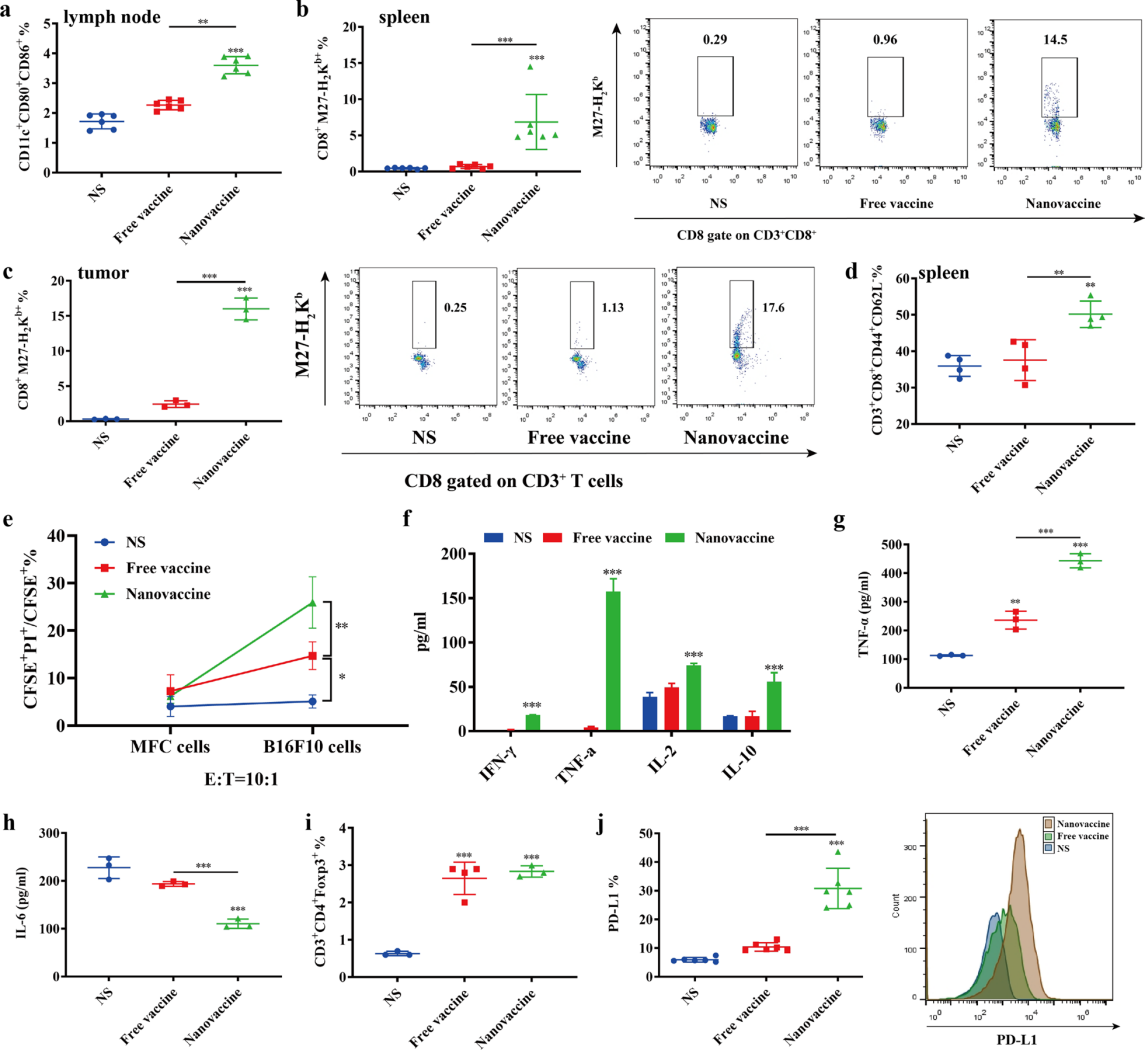
T cell responses activated by neoantigen nanovaccines. One week after last treatment, Proportions of mature DCs (CD11c^+^CD80^+^CD86^+^) in lymph nodes (**a**), proportions of neoantigen specific T cells (CD3^+^CD8^+^M27-H_2_K^b+^) in spleens (**b**) and tumors (**c**), and proportions of effector memory T cells (CD3^+^CD8^+^CD44^+^CD62L^−^) in spleens (**d**) were analyzed by flow cytometry. *P*-values were determined by one-way ANOVA with Tukey’s multiple comparisons test. **P = 0.0034 (**a**), **P = 0.0026 (d, NS vs Nanovaccine), **P = 0.0056 (d, Free vaccine vs Nanovaccine), ***P < 0.001. **e** Lymphocytes in spleens were incubated with CFSE labeled B16F10 melanoma cells and MFC forestomach cancer cells at effector-to-target ratio (E: T) of 10:1. PI was added 4 h after incubation and the percentage of dead tumor cells (CFSE^+^PI^+^/ CFSE^+^) was analyzed by flow cytometry. *P*-values were determined by one-way ANOVA with Tukey’s multiple comparisons test. *P = 0.0114 (NS vs Free vaccine), **P = 0.0046 (Free vaccine vs Nanovaccine). **f** Cytokines in the supernatant after co-incubation of lymphocytes and tumor cells. *P*-values were determined by two-way ANOVA with Tukey’s HSD multiple comparison post hoc test. ***P < 0.001. The level of TNF-α (**g**) and IL-6 (**h**) in the tumor microenvironment. **i** Proportions of regulatory T cells (CD3^+^CD4^+^Foxp3^+^) in the tumor microenvironment. **j** The expression of PD-L1 in tumors. *P*-values were determined by one-way ANOVA with Tukey’s multiple comparisons test. **P = 0.0014 (**g**), ***P < 0.001

To explore the anti-tumor mechanism of neoantigen nanovaccines, lymph nodes and spleens of mice in each group were excised to evaluate the systemic immune response one week after the last treatment. Compared with NS group, the proportion of mature DCs in lymph nodes and spleens was upregulated in other two groups (Fig. 5a). Especially in the nanovaccine group, the proportion of mature DCs was about 1.5-fold higher than that in the NS group. Accordingly, the neoantigen reactive T cells in the spleen and tumor of nanovaccine group were also significantly upregulated and reached 10-fold that of free vaccine group (Fig. 5b, c). Memory effector T cells (T_EM_, CD3^+^CD8^+^CD44^+^CD62L^−^) in nanovaccine group were also detected about 1.5-fold higher than NS group (Fig. 5d). To further verify the function of neoantigen reactive T cells, we examined the tumor-killing ability of spleen lymphocytes. Figure 5e shows that when effector target ratio (number of spleen lymphocytes: number of tumor cells) is 10:1, the proportion of dead tumor cells was the highest after incubation with spleen lymphocytes of nanovaccine group. The supernatant after co-incubation of lymphocytes and tumor cells was used to detect Th1 and Th2 cytokines. IL-2, IFN- γ and TNF-α, mainly secreted by Th1 cells, showed significant upregulation in nanovaccine group (Fig. 5f). IL-10 mainly secreted by Th2 cells was also slightly upregulated in nanovaccine group. In the tumor microenvironment, TNF-α was upregulated and IL-6 was down-regulated significantly in nanovaccine group (Fig. 5g, h).

In addition, the neoantigen nanovaccine also upregulated the Treg subgroup (CD3^+^CD4^+^Foxp3^+^) and the expression of PD-L1 on tumor cells (Fig. 5i, j). The above results show that the neoantigen nanovaccines may synergy with anti-PD1 antibody or Tregs inhibiting peptide P60 to treat post-surgical melanoma.

### Combination therapy

**Fig. 6:**
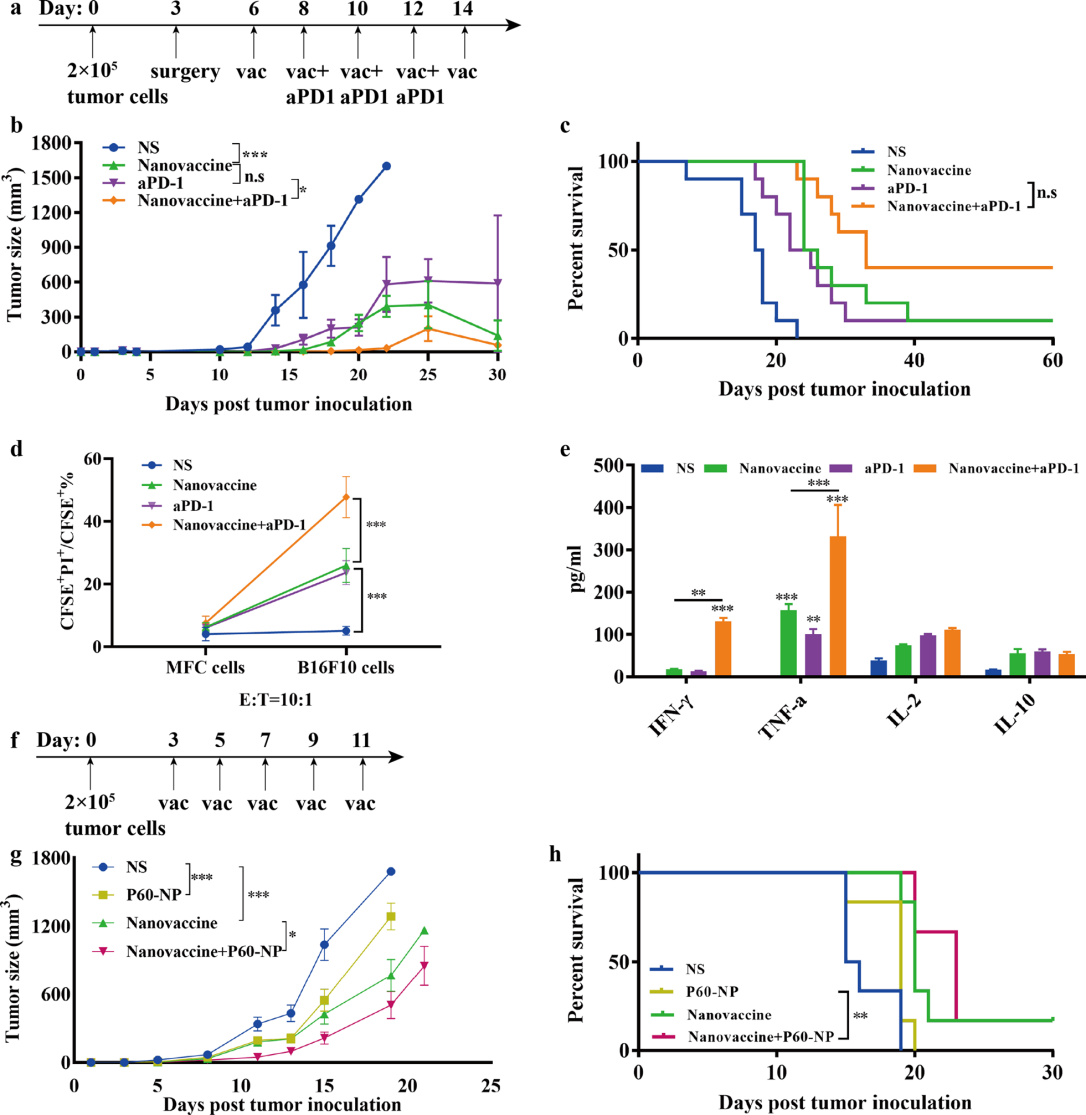
Neoantigen nanovaccines synergy with PD-1 blockade or Tregs targeting peptide to inhibit tumors. **a** Treatment schema of neoantigen nanocaccines combined with anti-PD1 antibody. C57BL/6 mice were implanted with B16F10 melanoma cells (2 × 10^5^) on the left lower abdomen on Day 0. About 3 days later, when the tumor size was almost 50 mm^3^, the tumor was surgically removed (surgical margin: tumor-cell positive). Mice received vaccine treatments on Day 6, 8, 10, 12 and 14, and anti-PD1 antibody treatments on Day 8, 10 and 12. **b** Growth curves represent the average tumor volumes of each group (n = 10). *P*-values were determined by two-way ANOVA with Tukey’s HSD multiple comparison post hoc test. ***P < 0.001, *P = 0.0461 (aPD1 vs Nanovaccine + aPD1). **c** Survival curves. (n = 10). *P*-values were determined by Log-rank (Mantel-Cox) test. *P = 0.0160 (aPD1 vs Nanovaccine + aPD1). **d** Lymphocytes in spleens were incubated with CFSE labeled B16F10 melanoma cells and MFC forestomach cancer cells at effector-to-target ratio (E: T) of 10:1. PI was added 4 h after incubation and the percentage of dead tumor cells (CFSE^+^PI^+^/ CFSE^+^) was analyzed by flow cytometry. *P*-values were determined by one-way ANOVA with Tukey’s multiple comparisons test. ***P < 0.001. **e** Cytokines in the supernatant after co-incubation of lymphocytes and tumor cells. *P*-values were determined by two-way ANOVA with Tukey’s HSD multiple comparison post hoc test. **P = 0.0015 (IFN-γ), **P = 0.0051 (TNF-α), ***P < 0.001. **f** Treatment schema of neoantigen nanocaccines combined with P60 nanoparticles. C57BL/6 mice were implanted with B16F10 melanoma cells (2 × 10^5^) on the left lower abdomen on Day 0, and received treatments on Day 3, 5, 7, 9 and 11. **g** Growth curves represent the average tumor volumes of each group. (n = 6). *P*-values were determined by two-way ANOVA with Tukey’s HSD multiple comparison post hoc test. ***P < 0.001, *P = 0.0248 (Nanovaccine vs Nanovaccine + P60-NP). **h** Survival curves (n = 6). *P*-values were determined by Log-rank (Mantel-Cox) test. **P = 0.0029 (P60-NP vs Nanovaccine + P60-NP)

In order to investigate the efficacy of neoantigen nanovaccines combined with anti-PD1 antibody, we established a recurrence-prevention mouse model as before. Mice after surgery were randomly divided into NS group, nanovaccine group, aPD-1 group and nanovaccine + aPD-1 group for treatment. The treatment scheme is shown in Fig. 6a. The average recurrence time in nanovaccine + aPD-1 group is about 3 days longer than that of nanovaccine group (19 vs 16, *p* = 0.2172), and 3.33 days longer than that of aPD-1 group (19 vs 15.4, *p* = 0.1020, Fig. 6b). Accordingly, the median survival time of mice in the nanovaccine + aPD-1 group was also prolonged 8 days than that of nanovaccine group (33 vs 25, *p* = 0.0982), and 9.5 days than that of aPD-1 group (33 vs 23.5, *p* = 0.016, Fig. 6c). Figure 6d shows that compared with the nanovaccine group, the proportion of dead tumor cells increased to 2-fold after incubation with spleen lymphocytes of mice in the nanovaccine + aPD-1 group. We also detected Th1 and Th2 cytokines in the supernatant as before. Upregulation of IL-2, IFN- γ and TNF-α was detected in the nanovaccine + aPD-1group, compared with nanovaccine group (Fig. 6e).

In addition, elimination of Tregs is also a key factor to improve the efficiency of cancer vaccines. A simple melanoma-bearing mouse model was established to evaluate the efficacy of neoantigen nanovaccines added with P60 nanoparticles (Fig. 6f). Compared with nanovaccine group, although nanovaccine + P60-NP group did not show significantly prolonged survival, the tumor growth rate became slower (Fig. 6g, h).

## Discussion

Effective antitumor immunity has been found to be related to the existence of T cells targeting cancer neoantigens, produced by tumor specific mutations and bypassed central thymus tolerance. Increased immune cell infiltration was observed in tumors with high neoantigen load [23], which was associated with good prognosis and benefit of ICIs in patients with melanoma [24, 25]. Three small single-arm studies where patients with melanoma received a neoantigen-based DC vaccine, an mRNA vaccine or a peptide vaccine, indicate that neoantigen vaccines can safely induce and amplify anti-tumor T cell responses [7, 26, 27].

The lymph node-delivery efficiency of nanoparticles may depend on a series of factors: particle size, shape, carrier material, charge and surface modification [28]. Studies have shown that small nanoparticles (particle size about 10–100 nm) can passively enter the lymph nodes through the afferent lymphatic vessels [29] and nanoparticles with a diameter of 20 nm or 45 nm reach more to lymph nodes and stay longer than that of 100 nm [30]. Due to smaller contact angles, spherical nanoparticles have higher APC uptake efficiency than worm-like ones [31]. In addition, negatively charged nanoparticles accumulate more easily in lymph nodes than neutral and positively charged nanoparticles because of less electrostatic interaction with the matrix. Through fluorescence images, we found that our nanovaccine with particle size about 24 nm (Fig. 2c, d), trafficked lymph nodes more than free vaccines (Fig. 3a–c). DCs are critical for presenting antigens to lymph node-resident T cells and the obvious co-localization signal of DCs and nanovaccines in lymph nodes (Fig. 3e) suggests that our nanovaccines can be ingested by DCs to initiate T cell responses.

Compared with the free vaccine, our neoantigen nanovaccine has obvious advantages: (1) more antigen accumulation in lymph nodes and higher proportion of activated DCs (Fig. 5a); (2) ten-fold the proportion of both systemic and tumor-infiltrating neoantigen specific T cells (Fig. 5b, c); (3) stronger toxicity of lymphocytes and higher secretion level of Th1/ Th2 cytokines (Fig. 5e, f). The production of neoantigen specific T cells is the key to successful work by neoantigen vaccines. Th1 cells mediated cellular immune response by secreting IL-2, IFN-γ and TNF-α. Th2 cells regulated humoral immune response by secreting IL-10. The effects of IL-10 on tumors are paradoxical [32]. It can inhibit antigen presentation and production of proinflammatory cytokines from APCs, and promote the survival and action of Foxp3^+^ Tregs, but also may contribute to the promotion of CD8^+^ T cell cytolytic function. When we examined the tumor-killing ability of spleen lymphocytes in vitro, the upregulation of IL-10 in both nanovaccine and nanovaccine + aPD-1group (Fig. 5e, f) with increased cytolytic effect on tumor cells. PD-1/PD-L1 pathway could promote the differentiation of CD4^+^ T cells to Tregs [33]. Since IL-10 cytokines are mainly produced by Th2 cells, Tregs and regulatory B cells, PD-1 antibody may slightly decrease the secretion of IL-10 through inhibiting Treg differentiation. Hence, compared to the nanovaccine group, there was no difference and even a decrease of IL-10 secretion in nanovaccine + aPD-1group. Interestingly, TNF-α was upregulated while IL-6 was downregulated in the tumor microenvironment. More and more evidence showed that IL-6 cytokine family, especially IL-6 and IL-11, promoted tumorigenesis and metastasis through IL-6 signaling pathway [34, 35].

The process of cancer vaccine initiating the immune system is mainly the process of antigen presentation. DCs encounter antigens at the injection site (or for DC vaccines, the antigen may be loaded on DCs before injection), and transport to lymph nodes, where T cells are activated. Mature DCs present tumor associated antigens on MHC class I and MHC class II molecules to CD8^+^ T cells (CTLs) and CD4^+^ T cells (Th cells) with immature or memory phenotypes. Cytokines produced by DCs also contribute to the production and expansion of activated tumor specific CD8^+^ and CD4^+^ T cell populations. T cells then transport to the tumor site and kill tumor cells through cytotoxicity against homologous antigens and secretion of cytokines such as IFN-γ and TNF-α. Finally, the killed tumor cells release a large amount of tumor antigens, which can be captured, processed and presented by APCs to induce polyclonal T cell responses, so as to increase the breadth and intensity of anti-tumor immune responses [36].

In this setting, after the treatment of our neoantigen cancer vaccine, the average recurrence time was extended from 11 days (control) to 16 days and the median survival time was even prolonged 7.5 days than that of NS group (Fig. 4b, c, e). These results were similar with other neoantigen nanovaccines with lymph-node-targeting characteristic, such as the vaccine nanodisc [37] and PEGylated reduced graphene oxide nanosheet [38] listed in the Additional file 1: Table S1. All vaccines loaded with neoantigens, which could induce powerful neoantigen specific T cell responses, achieved greater tumor-inhibition effects. But, compared to the vaccine nanodisc and nanosheet, our synthetic process is more simple and safe, with greater potential of clinical translation.

However, many inhibitory molecules and cells in the tumor microenvironment were upregulated under the immune pressure of cancer vaccines, such as Tregs and PD-L1 (Fig. 5i, j). In the clinical trial of a neoantigen RNA vaccine, Ugur Sahin et al. found that PD-L1 in tumor tissues was upregulated after vaccination as well [7]. Blocking PD1 was reported to be capable of enhancing the efficacy of cancer vaccines by inhibiting TGF-β and retinoic acid induced Tregs in the tumor microenvironment [39]. Therefore, we considered combining anti-PD1 antibody to eliminate the inhibitory effect of tumor microenvironment on T cells and improve the antitumor effect of neoantigen nanovaccines. The efficacy of the combined treatment group was significantly better than that of the vaccine or anti-PD1 antibody group alone (Fig. 6b, c). Moreover, after adding anti-PD1 antibody, T cells in spleens have stronger ability to kill tumor cells, indicating the systemic anti-tumor immune response is more effectively activated (Fig. 6d, e). It is suggested that this neoantigen nanovaccine and anti-PD1 antibody have synergistic antitumor effect, which provides a potential combined treatment strategy for patients with advanced tumors. Currently several clinical trials (NCT03639714, NCT03223103, NCT02721043, NCT04163094, NCT04015700 and NCT04251117) are exploring the combined efficacy of neoantigen vaccines and ICIs.

Local accumulation of immunosuppressive cells such as Tregs is linked to tumor immunoresistance via expression of inhibitory receptors, production of immunosuppressive cytokines and suppression of T cell functions [40]. An increase in the numbers of Tregs has reported to inhibit the efficacy of anti-PDL1 antibody [41] and associated with progression of a melanoma patient after CHP-NY-ESO-1 vaccination [42]. Depletion of Tregs could enhance anti-tumor immune responses by abrogating immunological unresponsiveness to syngeneic tumors [43]. Foxp3 is an important factor for the Treg, associated with its function [44]. P60 was able to inhibit nuclear translocation of Foxp3 and released the inhibition of the transcription factors NF-κB and NFAT [45, 46]. We also tried adding P60 to make nanovaccines and found a slight improvement of efficacy (Fig. 6f). In the future, with deeper knowledge of shared neoantigens and the tumor microenvironment, our vaccine platform can be further improved and applied into a variety of solid tumors.

## Conclusion

Overall, we developed a novel neoantigen nanovaccine based on PEG phospholipid derivatives and neo-peptides with strong tumor specificity and immunogenicity. This nanovaccine strategy targeting lymph nodes, can deliver neoantigens to DCs, and activate the tumor specific T cell immune responses more efficiently. The anti-tumor effect and safety of the neoantigen nanovaccine have been verified in melanoma mouse models, indicating its great potential for clinical translation. In addition, the combination of the neoantigen nanovaccine and anti-PD1 antibody or Treg inhibiting peptide P60 can further enhance the tumor inhibition effect, providing a feasible combination strategy for tumor immunotherapy.

## Supplementary Information

**Additional file 1: Figure S1.** Characterization of neoantigen nanoparticles. (a) MALDI-TOF–MS of DSPE-PEG2000-peptide (left: Tyrp1; right: M20). (c) Size, PDI, encapsulation efficiency, drug loading content and zeta-potential of three nanoparticles. **Table S1.** Comparison with model melanoma vaccines in the literature.

## Data Availability

All data relevant to the study are included in the article.

## Abbreviations

ICIs: Immune checkpoint inhibitors
ORR: Objective response rate
FDA: Food and drug administration
APCs: Antigen presenting cells
MHC: Major histocompatibility complex
PD-L1: Programmed cell death protein 1 ligand 1
CTLA-4: Cytotoxic T-lymphocyte-associated protein 4
Tregs: Regulatory T cells
MDSCs: Myeloid-derived suppressor cells
TILs: Tumor infiltrating lymphocytes
Foxp3: Forkhead box protein P3
DCs: Dendritic cells
SPF: Specific pathogen-free
FBS: Fetal bovine serum
PBS: Phosphate buffer saline
TEM: Transmission electron microscope
HPLC: High performance liquid chromatography
OVA: Ovalbumin
BMDCs: Bone marrow derived dendritic cells
HE: Hematoxylin–eosin
PD1: Programmed cell death protein 1
IL: Interleukin
mAbs: Monoclonal antibodies
T_EM_: Memory effector T cells

## References

[1] Hodi FS, O'Day SJ, McDermott DF (2010). Improved survival with ipilimumab in patients with metastatic melanoma. N Engl J Med.

[2] Hamid O, Robert C, Daud A (2019). Five-year survival outcomes for patients with advanced melanoma treated with pembrolizumab in KEYNOTE-001. Ann Oncol.

[3] Hu Z, Ott PA, Wu CJ (2018). Towards personalized, tumour-specific, therapeutic vaccines for cancer. Nat Rev Immunol.

[4] Kantoff PW, Higano CS, Shore ND (2010). Sipuleucel-T immunotherapy for castration-resistant prostate cancer. N Engl J Med.

[5] Chu Y, Liu Q, Wei J, Liu B (2018). Personalized cancer neoantigen vaccines come of age. Theranostics.

[6] Schumacher TN, Schreiber RD (2015). Neoantigens in cancer immunotherapy. Science (New York, NY).

[7] Sahin U, Derhovanessian E, Miller M (2017). Personalized RNA mutanome vaccines mobilize poly-specific therapeutic immunity against cancer. Nature.

[8] Keskin DB, Anandappa AJ, Sun J (2019). Neoantigen vaccine generates intratumoral T cell responses in phase Ib glioblastoma trial. Nature.

[9] Ott PA, Hu-Lieskovan S, Chmielowski B (2020). A phase Ib trial of personalized neoantigen therapy plus anti-PD-1 in patients with advanced melanoma, non-small cell lung cancer, or bladder cancer. Cell.

[10] Tejeda-Mansir A, García-Rendón A, Guerrero-Germán P (2019). Plasmid-DNA lipid and polymeric nanovaccines: a new strategic in vaccines development. Biotechnol Genet Eng Rev.

[11] Mellman I, Steinman RM (2001). Dendritic cells: specialized and regulated antigen processing machines. Cell.

[12] Guermonprez P, Saveanu L, Kleijmeer M, Davoust J, Van Endert P, Amigorena S (2003). ER-phagosome fusion defines an MHC class I cross-presentation compartment in dendritic cells. Nature.

[13] Burgdorf S, Schölz C, Kautz A, Tampé R, Kurts C (2008). Spatial and mechanistic separation of cross-presentation and endogenous antigen presentation. Nat Immunol.

[14] Leach DR, Krummel MF, Allison JP (1996). Enhancement of antitumor immunity by CTLA-4 blockade. Science (New York, NY).

[15] Iwai Y, Ishida M, Tanaka Y, Okazaki T, Honjo T, Minato N (2002). Involvement of PD-L1 on tumor cells in the escape from host immune system and tumor immunotherapy by PD-L1 blockade. Proc Natl Acad Sci USA.

[16] Morganti S, Curigliano G (2020). Combinations using checkpoint blockade to overcome resistance. Ecancermedicalscience.

[17] Ott PA, Hodi FS, Kaufman HL, Wigginton JM, Wolchok JD (2017). Combination immunotherapy: a road map. J Immunother Cancer.

[18] Hegde PS, Karanikas V, Evers S (2016). The where, the when, and the how of immune monitoring for cancer immunotherapies in the era of checkpoint inhibition. Clin Cancer Res.

[19] Casares N, Rudilla F, Arribillaga L (2010). A peptide inhibitor of FOXP3 impairs regulatory T cell activity and improves vaccine efficacy in mice. J Immunol (Baltimore, Md: 1950)..

[20] Kenter GG, Welters MJ, Valentijn AR (2009). Vaccination against HPV-16 oncoproteins for vulvar intraepithelial neoplasia. N Engl J Med.

[21] Aucouturier J, Dupuis L, Deville S, Ascarateil S, Ganne V (2002). Montanide ISA 720 and 51: a new generation of water in oil emulsions as adjuvants for human vaccines. Expert Rev Vaccines.

[22] Fenstermaker RA, Ciesielski MJ (2016). Clinical study of a survivin long peptide vaccine (SurVaxM) in patients with recurrent malignant glioma. Cancer Immunol Immunother..

[23] Brown SD, Warren RL, Gibb EA (2014). Neo-antigens predicted by tumor genome meta-analysis correlate with increased patient survival. Genome Res.

[24] Chan TA, Wolchok JD, Snyder A (2015). Genetic basis for clinical response to CTLA-4 blockade in Melanoma. N Engl J Med.

[25] Van Allen EM, Miao D, Schilling B (2015). Genomic correlates of response to CTLA-4 blockade in metastatic melanoma. Science.

[26] Carreno BM, Magrini V, Becker-Hapak M (2015). Cancer immunotherapy. A dendritic cell vaccine increases the breadth and diversity of melanoma neoantigen-specific T cells. Science..

[27] Ott PA, Hu Z, Keskin DB (2017). An immunogenic personal neoantigen vaccine for patients with melanoma. Nature.

[28] Ke X, Howard GP, Tang H (2019). Physical and chemical profiles of nanoparticles for lymphatic targeting. Adv Drug Deliv Rev.

[29] Irvine DJ, Swartz MA, Szeto GL (2013). Engineering synthetic vaccines using cues from natural immunity. Nat Mater.

[30] Reddy ST, Rehor A, Schmoekel HG, Hubbell JA, Swartz MA (2006). In vivo targeting of dendritic cells in lymph nodes with poly(propylene sulfide) nanoparticles. J Controlled Release.

[31] Champion JA, Mitragotri S (2006). Role of target geometry in phagocytosis. Proc Natl Acad Sci USA.

[32] Ouyang W, O'Garra A (2019). IL-10 family cytokines IL-10 and IL-22: from basic science to clinical translation. Immunity.

[33] Zhang Y, Liu Z, Tian M (2018). The altered PD-1/PD-L1 pathway delivers the 'one-two punch' effects to promote the Treg/Th17 imbalance in pre-eclampsia. Cell Mol Immunol.

[34] Taniguchi K, Karin M (2014). IL-6 and related cytokines as the critical lynchpins between inflammation and cancer. Semin Immunol.

[35] Johnson DE, O'Keefe RA, Grandis JR (2018). Targeting the IL-6/JAK/STAT3 signalling axis in cancer. Nat Rev Clin Oncol.

[36] Saxena M, van der Burg SH, Melief CJM, Bhardwaj N (2021). Therapeutic cancer vaccines. Nat Rev Cancer.

[37] Kuai R, Ochyl LJ, Bahjat KS, Schwendeman A, Moon JJ (2017). Designer vaccine nanodiscs for personalized cancer immunotherapy. Nat Mater.

[38] Xu C, Hong H, Lee Y (2020). Efficient lymph node-targeted delivery of personalized cancer vaccines with reactive oxygen species-inducing reduced graphene oxide nanosheets. ACS Nano.

[39] Dyck L, Wilk MM, Raverdeau M, Misiak A, Boon L, Mills KH (2016). Anti-PD-1 inhibits Foxp3(+) Treg cell conversion and unleashes intratumoural effector T cells thereby enhancing the efficacy of a cancer vaccine in a mouse model. Cancer Immunol Immunotherapy.

[40] Saleh R, Elkord E (2019). Treg-mediated acquired resistance to immune checkpoint inhibitors. Cancer Lett.

[41] Oweida A, Hararah MK, Phan A (2018). Resistance to radiotherapy and PD-L1 blockade is mediated by TIM-3 upregulation and regulatory T-Cell infiltration. Clin Cancer Res.

[42] Tsuji K, Hamada T, Uenaka A (2008). Induction of immune response against NY-ESO-1 by CHP-NY-ESO-1 vaccination and immune regulation in a melanoma patient. Cancer Immunol Immunotherapy.

[43] Yamaguchi T, Sakaguchi S (2006). Regulatory T cells in immune surveillance and treatment of cancer. Semin Cancer Biol.

[44] Lozano T, Gorraiz M, Lasarte-Cía A (2017). Blockage of FOXP3 transcription factor dimerization and FOXP3/AML1 interaction inhibits T regulatory cell activity: sequence optimization of a peptide inhibitor. Oncotarget.

[45] Setiawan MF, Rudan O, Vogt A (2019). FOXP3 inhibitory peptide P60 increases efficacy of cytokine-induced killer cells against renal and pancreatic cancer cells. Anticancer Res.

[46] Ding X, Peng C, Li Y (2020). Targeting inhibition of Foxp3 by MMP2/9 sensitive short peptide linked P60 fusion protein 6(P60-MMPs) to enhance antitumor immunity. Macromol. Biosci..

