# Supplementary material for: Lymph node-targeted neoantigen nanovaccines potentiate anti-tumor immune responses of post-surgical melanoma
Source: J Nanobiotechnology. 2022 Apr 13;20:190. doi: 10.1186/s12951-022-01397-7 (PMC9006542; doi:10.1186/s12951-022-01397-7)
Supplement: Supplementary file 1 — Additional file 1: Figure S1. Characterization of neoantigen nanoparticles. (a) MALDI-TOF–MS of DSPE-PEG2000-peptide (left: Tyrp1; right: M20). (c) Size, PDI, encapsulation efficiency, drug loading content and zeta-potential of three nanoparticles. Table S1. Comparison with model melanoma vaccines in the literature. [file 12951_2022_1397_MOESM1_ESM.docx]

Supplementary Materials for

Lymph node-targeted neoantigen nanovaccines potentiate anti-tumor immune responses of post-surgical melanoma.

Yanhong Chu^1^, Lingyu Qian^1, 2^, Yaohua Ke^1^, Xiaoyu Feng^1^, Xinjie Chen^1^, Fangcen Liu^3^, Lixia Yu^1^, Lianru Zhang^1^, Yaping Tao^1^, Rui Xu^1^, Jia Wei^1^, Baorui Liu^1^, Qin Liu^1,*^.


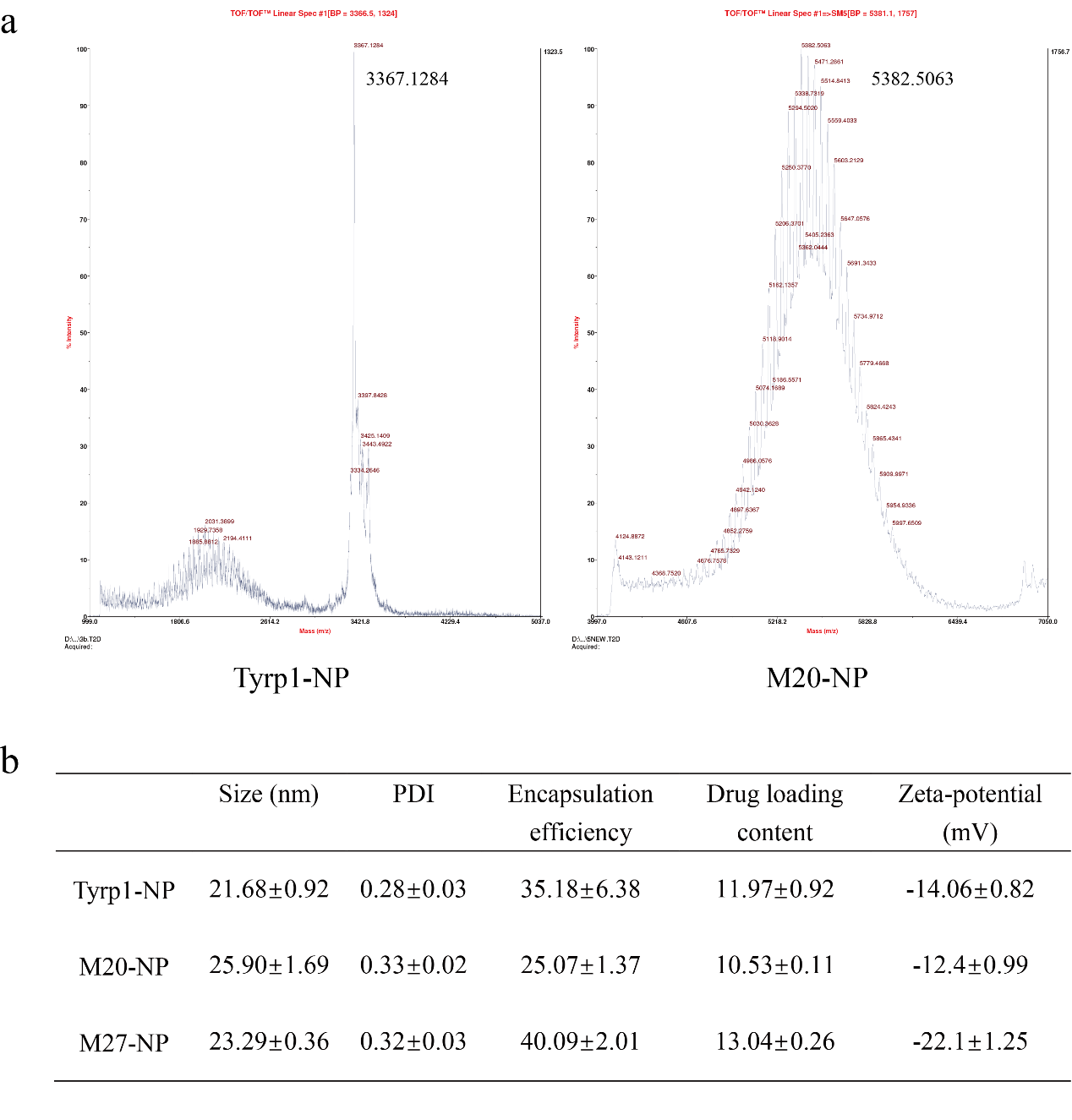


**Additional file 1: Figure S1. Characterization of neoantigen nanoparticles.** (a) MALDI-TOF-MS of DSPE-PEG_2000_-peptide (left: Tyrp1; right: M20). (c) Size, PDI, encapsulation efficiency, drug loading content and zeta-potential of three nanoparticles.

Additional file 1: Table S1. Comparison with model melanoma vaccines in the literature.

| Antigen (amount) | Vehicle | Adjuvant | Injection | Immune Responses | Combination Therapy |
| --- | --- | --- | --- | --- | --- |
| neoantigens (2)  tumor associated antigens (1) | DSPE-PEG_2000_ | Montanide^TM^ ISA 51 | subcutaneous | neoantigen specific T cell responses | anti PD-1 antibody,  Treg inhibiting peptide P60 |
| neoantigens (2)  tumor associated antigens (1) [1] | phospholipids and apolipoprotein-1 mimetic peptides | CpG | subcutaneous | neoantigen specific T cell responses | anti PD-1 antibody,  anti CTLA-4 antibody |
| Neoantigen (2) [2] | reduced graphene oxide nanosheets | CpG | subcutaneous | neoantigen specific T cell responses | anti PD-1 antibody |
| OVA, tumor cell membranes [4] | fluoropolymers | fluoropolymers | subcutaneous | antigen specific T cell responses | anti PD-1 antibody,  anti CTLA-4 antibody |
| OVA [5] | PC7A copolymer | PC7A | subcutaneous | STING-dependent adaptive immune responses | anti PD-1 antibody |

References:

1. Rui Kuai, Lukasz J Ochyl, Keith S Bahjat, Anna Schwendeman, James J Moon. Designer vaccine nanodiscs for personalized cancer immunotherapy. Nature Material. 2017; 16(4):489-496.

2. Xu C, Hong H, Lee Y, et al. Efficient Lymph Node-Targeted Delivery of Personalized Cancer Vaccines with Reactive Oxygen Species-Inducing Reduced Graphene Oxide Nanosheets. ACS nano. 2020;14(10):13268-78.

3. Jun Xu, Jia Lv, Qi Zhuang, Zongjin Yang, Zhiqin Cao, Ligeng Xu, et, al. A general strategy towards personalized nanovaccines based on fluoropolymers for post-surgical cancer immunotherapy. Nature Nanotechnology. 2020; 15(12):1043-1052.

4. Min Luo, Hua Wang, Zhaohui Wang, Haocheng Cai, Zhigang Lu, Yang Li, et, al. A STING-activating nanovaccine for cancer immunotherapy. Nature Nanotechnology. 2017; 12(7):648-654.
